# Prevalence of and risk factors for asthma among people aged 45 and older in China: a cross-sectional study

**DOI:** 10.1186/s12890-021-01664-7

**Published:** 2021-10-04

**Authors:** Jingxuan Wan, Qing Zhang, Chunxiao Li, Jiangtao Lin

**Affiliations:** 1 Graduate School of Peking Union Medical College, Chinese Academy of Medical Sciences/Peking Union Medical College, Beijing, China; 2 Department of Pulmonary and Critical Care Medicine, Center of Respiratory Medicine, China-Japan Friendship Hospital, Beijing, China; 3 Peking University Health Science Center, Beijing, China

**Keywords:** Asthma, Prevalence, Trends, Epidemiology, CHARLS, Risk factors, China

## Abstract

**Background:**

Asthma is one of the most prevalent chronic respiratory diseases worldwide. This study aimed to determine the updated prevalence of and risk factors for asthma among individuals aged 45 and older in mainland China.

**Methods:**

The data for this study came from the fourth wave of the China Health and Retirement Longitudinal Study (CHARLS) conducted by the National School of Development of Peking University in 2018. The CHARLS is a nationally representative survey targeting populations aged 45 and over from 28 provinces/cities in mainland China. A representative sample of 19,816 participants was recruited for the study using a multistage stratified sampling method. The prevalence of asthma was determined across different characteristics. The potential risk factors were examined by multivariable logistic regressions.

**Results:**

A total of 18,395 participants (8744 men and 9651 women) were eligible for the final data analysis. The estimated prevalence of asthma among Chinese people aged ≥ 45 years in 2018 was 2.16% (95% CI 1.96–2.38). The prevalence of asthma significantly differed according to race (*P* = 0.002), with an overall rate of 2.07% (95% CI 1.86–2.29) in Han paticipants and 3.32% (95% CI 2.50–4.38) in minority participants. Furthermore, the minority ethnicities (OR = 1.55 [95% CI 1.12–2.14], *P* = 0.008), older age (60–69 years group: OR = 1.85 [95% CI 1.17–2.92], *P* = 0.008; ≥ 70 years group: OR = 2.63 [95% CI 1.66–4.17], *P* < 0.001), an education level of middle school or below (middle-school education: OR = 1.88 [95% CI 1.15–3.05], *P* = 0.011; primary education: OR = 2.48 [95% CI 1.55–3.98], *P* < 0.001; literate: OR = 2.53 [95% Cl 1.57–4.07], *P* < 0.001; illiterate: OR = 2.78 [95% CI 1.72–4.49, *P* < 0.001]), smoking (OR = 1.37 [95% CI 1.11–1.68], *P* = 0.003), and residence in North (OR = 1.52 [95% CI 1.11–2.09], *P* = 0.01) or Northwest China (OR = 1.71 [95% CI 1.18–2.49], *P* = 0.005) were associated with prevalent asthma.

**Conclusions:**

Asthma is prevalent but underappreciated among middle-aged and elderly people in China. A number of risk factors were identified. These results can help to formulate correct prevention and treatment measures for asthma patients.

## Background

Asthma is one of the most prevalent chronic respiratory disorders and is a major cause of disability, health resource utilization and socioeconomic burden worldwide [1–3]. The World Health Survey (WHS) reported that the global prevalence rate of doctor-diagnosed asthma among individuals aged 18–45 years was 4.3% in 2002–2003 [4]. The Global Initiative for Asthma (GINA) Dissemination Committee Report estimated that asthma affected 300 million people worldwide in 2004 [5]. Acooding to the Global Burden of Disease (GBD) study in 2015, the population with asthma worldwide increased to 358 million, and the number is predicted to rise to 400 million by 2025 [5]. Obviously, with the rapid development of global industrialization and urbanization and changes in the ecological environment, the prevalence of asthma has shown an upward trend.

In China, the China Asthma and Risk factors Epidemiologic (CARE) study reported a physician-diagnosed asthma prevalence of 1.2% for people aged ≥ 14 years from eight provinces/cities in 2012 [6]. The China Pulmonary Health (CHP) study from 2015 reported this prevalence to be 4.2% in adults aged ≥ 20 years based on wheezing symptoms in the preceding 12 months or previous diagnosis by a physician from ten provinces/cities [7]. Differences in the diagnosis of asthma and data collection might contribute to this inconsistency. Overall, much attention has focused on childhood and adult asthma [8, 9], but the population is ageing rapidly in China [10, 11], and many studies have demonstrated that the prevalence of asthma increases with age [7]. Therefore, it is necessary to describe the prevalence of asthma in middle-aged and elderly people. However, current studies are not up to date and cannot reflect the recent prevalence of asthma among middle-aged and elderly people in China.

Here, we used data from the CHARLS survey to describe the latest prevalence of asthma in Chinese individuals aged 45 years and older in 2018 and to assess the risk factors for asthma.

## Methods

### Study design and population

**Fig. 1:**
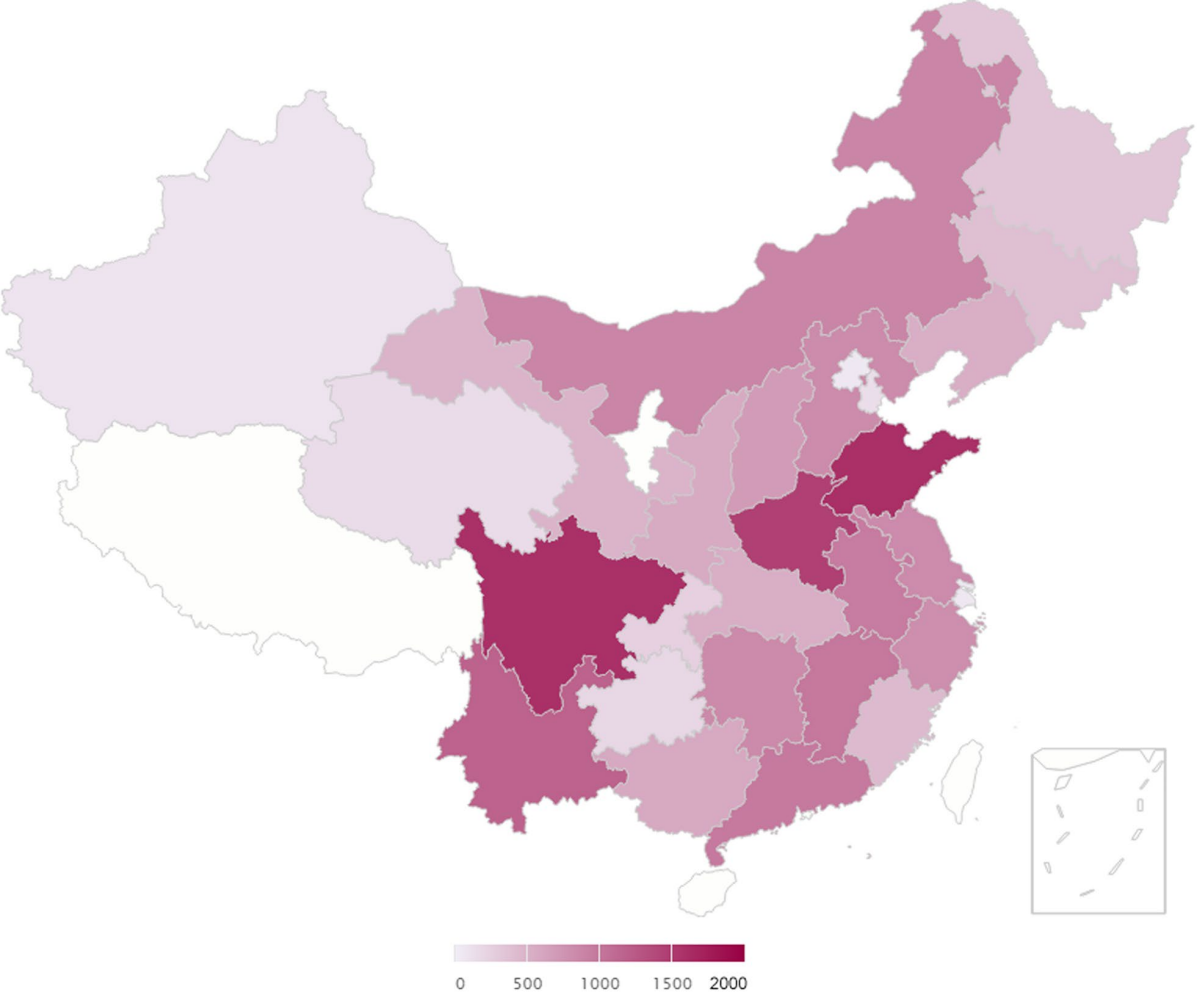
Geographic and population distribution of 28 provinces/cities in the CHARLS in 2018. Each province/city on the map is gradually darkened in color as the number of participants increases

Data for this study were obtained from the fourth wave of the CHARLS survey conducted by the National School of Development of Peking University in 2018. The details of the sampling design of this survey have been described previously [12]. Briefly, the CHARLS is a nationally representative survey targeting populations aged 45 and older from 450 villages/urban communities in 150 counties/districts in 28 provinces/cities in mainland China that provides information about demographics, geography, health status, and lifestyle variables of individuals. The study was carried out in 2018 with a response rate of 86%. The geographic and population distribution of 28 provinces/cities is presented in Fig. 1.

**Fig. 2:**
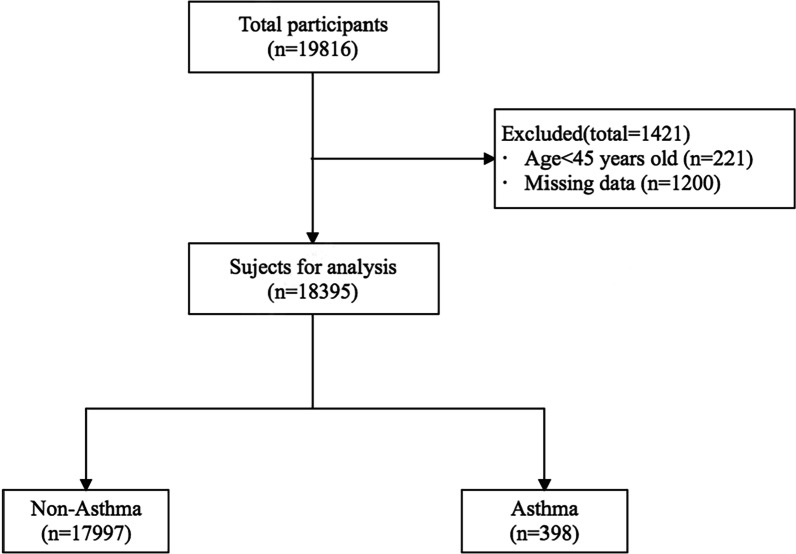
Flow chart for subjects enrolled in this study. Missing data refers to incompleteness of demographic/geographic/lifestyle information

Overall, a total of 19,816 participants were recruited for the CHARLS in 2018, of whom 1421 were excluded due to incomplete information or age < 45 years. Finally, 18,395 participants were enrolled (see Fig. 2). The Medical Ethics Committee approved the CHARLS, and all interviewees were required to sign an informed consent form. Ethics approval for the data collection in the CHARLS was obtained from the Biomedical Ethics Review Committee of Peking University (IRB00001052-11015). Ethics approval for the use of CHARLS data was obtained from the University of Newcastle Human Research Ethics Committee (H-2015-0290).

### Data collection

At each county or district unit, trained staff collected data according to a standard protocol in respondents’ homes and local health stations or the local office of the Chinese Center for Disease Prevention and Control (CDC). For each participant, an interview was conducted to collect information on sociodemographic characteristics (race, age, sex and education), residence setting, geographic region, and lifestyle features (smoking status, cooking fuels type).

According to self-reported physician diagnosis of asthma, paticipants were classified into two groups: the asthma and non-asthma groups. The participants’ race was classified as Han or Minority (Zhuang, Man, Hui, Miao, Weiwuer, Tujia,Yi, Mongol, Zangand or other). The participants were divided into four groups according to their age, namely 45–49 years old group, 50–59 years, 60–69 years, and ≥ 70 years old. Educational attainment was classified as illiterate (no formal education), literate (did not finish primary school but capable of reading or writing), primary education (graduated from elementary school), middle-school education (graduated from middle school) and high school education and above (graduated from high school, vocational school, college or post-graduate school). The participants’ residence was classified as urban, urban–rural area, rural and categorized into six geographic regions, namely, East China, North China, Northeast China, Northwest China, South Central China and Southwest China [13]. According to the participants’ smoking status, they were classified as non-smokers and smokers (smokers were defined as current smokers who had smoked more than 100 cigarettes in their life). Cooking fuel type was classified as clean (natural gas, marsh gas, liquefied petroleum gas or electricity) or unclean (coal, crop residue or wood burning).

### Quality control

Fieldwork was conducted by independent enumerators who collected data using face-to-face computer-assisted personal interviews. Data were verified by the CHARLS headquarters. Furthermore, the first two interviews were recorded to verify that participants performed the correct procedures. If the recording could not be completed owing to technical or other problems, CHARLS headquarters would call back for a phone check. The above technicians were trained to avoid information bias. Additionally, multistage stratified cluster sampling was used to resolve selection bias. Confounding factors were addressed with multivariable­adjusted logistic regression analysis.

### Statistical analysis

In this study, estimations of the prevalence of and risk factors for asthma were weighted by considering the study design and individual weight with individual and household non-response adjustment [14]. Metrical data with a normal distribution are presented as the mean ± standard deviation. Categorical data are presented as absolute counts and percentages. First, univariate analyses were performed using the chi‐square test. The Mantel–Haenszel chi-square test was used to test linear trends for ordered classification variables. Then, the variables with a *P* < 0.05 in univariate comparison were included in the multivariate logistic regression analysis to determine risk factors associated with asthma. Multivariate logistic regression was used to calculate the adjusted odds ratios (ORs). Asthma was the dependent variable, and the independent variables included race, age, education, geographic region, smoking status and cooking fuel type. Data cleaning and analysis were performed using Stata statistical software (version 15.0; Stata Corporation, College Station, TX, USA). A *P* value < 0.05 was regarded as statistically significant.

## Results

### Demographic

The study included 18,395 paticipannts (8744 men and 9651 women) aged 45 years and older who completed the survey. Of the total paticipants, 16,977 (92.3%) were Han Chinese, and 1418 (7.7%) were of other origins. Furthermore, 7752 (41.9%) paticipants were current smokers. The distribution of our study population by general characteristics is summarized in Table 1.

**Table 1:** General characteristics of the participants among aged 45 and older

Characteristic	Total	Asthma	Non-Asthma
Participants	18,395 (100%)	398 (2.16%)	17,997 (97.84%)
*Race*			
Han	16,977 (92.3%)	351 (88.2%)	16,626 (92.4%)
Minorities	1418 (7.7%)	47 (11.8%)	1371 (7.6%)
*Sex*			
Male	8744 (47.5%)	201 (50.5%)	8543 (47.5%)
Female	9651 (52.5%)	197(49.5%)	9454 (52.5%)
*Age group*			
45–49 years	1884 (10.2%)	22 (5.5%)	1862 (10.4%)
50–59 years	6326 (34.4%)	92 (23.1%)	6234 (34.6%)
60–69 years	6179 (33.6%)	146 (36.7%)	6033 (33.5%)
≥ 70 years	4006 (21.8%)	138 (34.7%)	3868 (21.5%)
*Education*			
High-school or above	2389 (13.0%)	22 (5.5%)	2367(13.2%)
Middle-school	4087 (22.2%)	67 (16.8%)	4020 (22.3%)
Primary education	4049 (22.0%)	97 (24.4%)	3952 (22.0%)
Literate	3828 (20.8%)	95 (23.9%)	3733 (20.7%)
Illiterate	4042 (22.0%)	117 (29.4%)	3925 (21.8%)
*Residence setting*			
Urban	3724 (20.3%)	70 (17.6%)	3654 (20.3%)
Urban–rural area	1498 (8.1%)	28 (7.0%)	1470 (8.2%)
Rural	13,173 (71.6%)	300 (75.4%)	12,873 (71.5%)
*Geographic region*			
Northeast	1274 (6.9%)	20 (5.0%)	1254 (7.0%)
East	5666 (30.8%)	106 (26.6%)	5560 (30.9%)
South Central	4387 (23.8%)	82 (20.6%)	4305(23.9%)
Southwest	3199 (17.4%)	80 (20.1%)	3119 (17.3%)
North	2572 (14.0%)	69 (17.4%)	2503 (13.9%)
Northwest	1297 (7.1%)	41 (10.3%)	1256 (7.0%)
*Smoking status*			
Non-smokers	10,643 (58.1%)	203 (51.1%)	10,440 (58.2%)
Smokers	7752 (41.9%)	195 (48.9%)	7557 (41.8%)
*Type of cooking*			
Clean	12,895 (70.1%)	252 (63.3%)	12,643 (70.3%)
Unclean	5500 (29.9%)	146 (36.7%)	5354 (29.7%)

Smokers were defined as current smokers who smoking more than 100 cigarettes in your life

### Prevalence of asthma in 2018

Of the 18,395 paticipants, 398 were diagnosed with asthma. The total prevalence of asthma in Chinese people aged 45 years and older was estimated to be 2.16% (95% CI 1.96–2.38).

### The prevalence of asthma according to different characteristics

Among people aged 45 and over in China, the prevalence of asthma in Han and other ethnic groups was 2.07% (95% CI 1.86–2.29) and 3.32% (95% CI 2.50–4.38), respectively (*P* = 0.002). The prevalence of asthma significantly increased with age, from 1.17% (95% CI 0.77–1.77) in individuals aged 45–59 years to 3.45% (95% CI 2.92–4.06, *P* < 0.001) in those aged 70 years and older. The prevalence of asthma significantly decreased with increasing education attainment, from 2.89% (95% CI 2.42–3.46) in adults who were illiterate to 0.92% (95% CI 0.61–1.39 *P* < 0.001) in those who were educated at the high school level or above. We found that the prevalence of asthma significantly varied between different geographic regions in China, ranging from 1.57% (95% CI 1.02–2.42) in Northeast China to 3.16 (95% CI 2.34–4.27) in Northwest China (*P* = 0.004). In non-smokers, the prevalence of asthma was 1.91% (95% CI 1.66–2.19), compared with 2.52% (95% CI 2.19–2.89) in smokers (*P* = 0.005). The prevalence of asthma was 1.95% (95% CI 1.73–2.20) for those who used clean cooking fuels and 2.65% (95% CI 2.26–3.11, *P* = 0.003) for those who used unclean cooking fuels. However, asthma prevalence did not differ between men (2.30% [95% CI 2.00–2.63]) and women (2.04% [95% CI 1.78–2.34], *P* = 0.231) or among participants living in urban (1.88% [95% CI 1.49–2.37]), urban–rural (1.87% [95% CI 1.29–2.69]) and rural (2.28% [95% CI 2.04–2.55], *P* = 0.242) areas. The prevalence of asthma stratified according to different characteristics is presented in Table 2.

**Table 2:** The prevalence of asthma by different characteristics among people aged 45 and older

Characteristic	Prevalence (%)	95% CI	*P*
*Race*			0.002^a^
Han	2.07	1.86–2.29	
Minorities	3.32	2.50–4.38	
*Sex*			0.231^a^
Male	2.30	2.00–2.63	
Female	2.04	1.78–2.34	
*Age group*			< 0.001^b^
45–49 years	1.17	0.77–1.77	
50–59 years	1.45	1.19–1.78	
60–69 years	2.36	2.01–2.77	
≥ 70 years	3.45	2.92–4.06	
*Education*			< 0.001^b^
High-school or above	0.92	0.61–1.39	
Middle-school	1.64	1.29–2.07	
Primary education	2.40	1.97–2.92	
Literate	2.48	2.03–3.03	
Illiterate	2.89	2.42–3.46	
*Residence setting*			0.242^b^
Urban	1.88	1.49–2.37	
Urban–rural area	1.87	1.29–2.69	
Rural	2.28	2.04–2.55	
*Geographic region*			< 0.001^b^
Northeast	1.57	1.02–2.42	
East	1.87	1.55–2.26	
South Central	1.87	1.51–2.32	
Southwest	2.50	2.01–3.10	
North	2.68	2.12–3.38	
Northwest	3.16	2.34–4.27	
*Smoking status*			0.005^a^
Non-smokers	1.91	1.66–2.19	
Smokers	2.52	2.19–2.89	
Type of cooking			0.003^a^
Clean	1.95	1.73–2.20	
Unclean	2.65	2.26–3.11	

The *P* value was calculated using Chi-square

*CI* confidence interval

^a^Chi-square test

^b^Mantel–Haenszel chi-square test

### Sociodemographic, geographic and lifestyle factors associated with asthma

These statistically significant variables in the univariate model including race, age, education, geographic region, smoking status, and cooking fuel type, were analysed in the multivariable analysis. Multivariable logistic regression analysis was performed to control for the effect of potentially confounding variables. Finally, the analysis showed that the risk factors for asthma include race, age, smoking and North or Northwest China residence, while a middle-school education and above is a protective factor for asthma. Individuals in ethnic minorities were demonstrated to be more at risk factor for asthma than Han people (OR = 1.55 [95% CI 1.12–2.14], *P* = 0.008). Those who aged 60 years or older had a higher risk of suffering from asthma than those who aged 45–49 years (60–69 years group: OR = 1.85 [95% CI 1.17–2.92], *P* = 0.008; ≥ 70 years group: OR = 2.63 [95% CI 1.66–4.17], *P* < 0.001). The prevalence of asthma was higher among smokers than non-smokers (OR = 1.37 [95% CI 1.11–1.68], *P* = 0.003). People living in North or Northwest China had a higher risk of asthma than those who living in East China (North China: OR = 1.52[95% CI 1.11–2.09], *P* = 0.01; Northwest China: OR = 1.71 [95% CI 1.18–2.49], *P* = 0.005). Additionally, participants with a middle-school education and below had a higher risk than those who were graduated from high school or above (middle-school education: OR = 1.88[95%CI 1.15–3.05], *P* = 0.011; primary education: OR = 2.48 [95% CI 1.55–3.98], *P* < 0.001; literate: OR = 2.53 [95% Cl 1.57–4.07], *P* < 0.001; illiterate: OR = 2.78 [95% CI 1.72–4.49], *P* < 0.001). There were no definite differences in asthma rates according to fuel type used for cooking. The multivariable results are presented in Table 3.

**Table 3:** Relationship of risk factors and the prevalence of asthma from the multivariable regression analysis

Risk factor	OR (95% CI)	*P*
*Race*		
Han (reference)	1.00	.
Minorities	1.55 (1.12–2.14)	0.008
*Age*		
45–49 years (reference)	1.00	.
50–59 years	1.24 (0.77–1.98)	0.374
60–69 years	1.85 (1.17–2.92)	0.008
≥ 70 years	2.63 (1.66–4.17)	< 0.001
*Education*		
High-school or above (reference)	1.00	.
Middle-school	1.88 (1.15–3.05)	0.011
Primary education	2.48 (1.55–3.98)	< 0.001
Literate	2.53 (1.57–4.07)	< 0.001
Illiterate	2.78(1.72–4.49)	< 0.001
*Geographic region*		
East (reference)	1.00	.
North	1.52 (1.11–2.09)	0.010
Northeast	0.86 (0.52–1.40)	0.532
Northwest	1.71 (1.18–2.49)	0.005
South Central	1.02 (0.76–1.37)	0.885
Southwest	1.16 (0.85–1.56)	0.349
*Smoking status*		
Non-smokers (reference)	1.00	.
Smokers	1.37 (1.11–1.68)	0.003
*Type of cooking*		
Clean (reference)	1.00	.
Unclean	1.07 (0.86–1.32)	0.564

These variables with a *P* < 0.05 in univariate comparison were included in the multivariate logistic regression analysis

*OR* odds ratio, *CI* confidence interval

## Discussion

Our large comprehensive asthma survey was administered to a nationally representative sample of Chinese individuals aged ≥ 45 years, and the results indicated that the total estimated prevalence of asthma in these individuals in China in 2018 was 2.16%. The prevalence of asthma was higher among individuals in ethnic minorities than among Han people (3.32% vs 2.07%). In addition, we observed that the prevalence of asthma was the highest in people aged ≥ 70 years (3.45%), who were illiterate (2.89%) and who lived in Northwest China (3.16%). Smokers were linked to a higher risk of asthma than non-smokers (2.52% vs 1.91%).

A cross-sectional survey based on the CHARLS of the prevalence of asthma-chronic obstructive pulmonary disease overlap (ACO) was published in 2011 [15]. However, some of these patients with asthma were classified as asthma-chronic obstructive pulmonary disease overlap groups, which led to a lower overall prevalence of asthma (1.33%). The CARE study was performed from 2010 to 2012, including 164,215 Chinese people aged > 14 years from eight provinces/cities (Shanghai, Guangdong, Beijing, Jiangsu, Sichuan, Henan, Liaoning and Shanxi), and estimated that the prevalence of asthma among individuals aged > 14 years in mainland China was 1.24%. Our study was a large-scale investigation that geographically covered 28 out of 31 provinces in mainland China. This study reported that the rate of asthma among people aged ≥ 45 years was 2.16%, which was similar to the value of 2.18% among people aged > 50 years reported by the CARE study [6]. Furthermore, the CHP study reported this prevalence to be 4.2% based on wheezing symptoms or physician diagnosis from ten provinces/cities in 2015, which was higher than our study. Although the demographic characteristics of the participants in these studies were not exactly matched, the higher prevalence reported by the CHP study may be partly explained by diagnostic criteria, including wheezing symptoms, which could potentially have led to the misclassification of chronic obstructive pulmonary disease(COPD) or cardiogenic lung disease as asthma as those diseases present with wheezing [7]. Additionally, in the middle-aged and elderly populations, the prevalence of asthma increases with age [6, 7, 15]. Given that China has the largest ageing population in the world and currently has one of the highest ageing rates in the world [10], it is essential to pay more attention to the asthma among people aged ≥ 45 years.

Multivariate analysis demonstrated that the primary risk factors for asthma among people aged ≥ 45 years were race, age (≥ 60 years), middle-school education and below, smoking, and residency in North and Northwest China residence. Data generated from countries with multi-ethnic populations presented significant differences in the relative rates of asthma by ethnicity. In the United States, for example, the prevalence of asthma among Hispanics, Caucasians, African Americans, and Puerto Ricans was 6.6%, 7.8%, 10.3%, and 13.7%, respectively [16–19]. UK data suggested that the prevalence of asthma was higher in those of African descent and lower in South Asians than in Caucasians [20, 21]. Moreover, by comparing immigrants of different ethnicities, Aarab et al. found that migration and other environmental factors might not have the same effect on all asthma phenotypes and that the prevalence of adult-onset asthma varies by ethnicity [22]. Genetic variations in susceptibility genes in specific populations have been shown previously [23, 24]. In our study, the Han ethnicity was significantly associated with a decreased prevalence of asthma. Previous research has shown that polymorphisms in the gene encoding for metalloprotease 33 (ADAM33) are closely associated with the risk of asthma attacks in different populations. Shufen Zhu et al. analysed the single nucleotide polymorphisms (SNPs) in the T1, T2 and V4 loci of the ADAM33 gene and found that polymorphisms in the T1 locus were significantly associated with asthma risk in both Mongolian and Han ethnicities and that polymorphisms in the V4 locus were relevant only in the Mongolian patients [25]. Thus, genetics may play a role in the link between asthma and ethnicity, although evidence is limited. Moraes et al. proposed that asthma is a heterogeneous disease caused by complex interactions between genetic and environmental factors [26]. Further research into the ethnicity-related causes of asthma is needed. Therefore, our recommendation is to include more detailed information about race in clinical and epidemiological studies.

Recent studies have suggested a relationship between asthma and age. Those studies indicated that the prevalence of asthma increased with age [6, 27–29], consistent with our study. Compared to people aged 45–49 years, the odds of asthma among people aged 60–69 years were approximately increased by approximately 1.85-fold, and odds for those over 70 were increased by 2.63-fold. In Korea, a survey of adult asthma incidence based on the National Health Insurance Service–National Sample Cohort (NHIS-NSC) observed that incidence rates increased with age, and participants aged 70 or over showed the highest asthma prevalence [30]. In Poland, age over 60 years was a significant risk factor for asthma occurrence [31]. The higher prevalence of asthma among older adults than younger individuals can be explained by the cumulative incidence of this disease. Asthma is still considered an incurable condition, and it can be diagnosed at any age. In addition to genetic determinants, the influence of environmental factors (exposure to allergens, chemical and biological air pollution, infections, obesity) also increases with age [31]. Furthermore, it is necessary to pay more attention to the aged and strengthen the care of asthma patients who are over 60 years old.

Substantial studies have been published showing that asthma is positively associated with smoking [6, 7, 31]. A case–control study found that in the population consisting of adults aged 21–63 years, the risk of asthma was higher among smokers, with an odds ratio of 1.33, than among non-smokers [32], which was similar to the odds ratio of 1.37 in our study, raising the question of why current smokers have a higher risk of asthma? Kim et al. reported that bronchial responsiveness (BHR) is positively associated with active smoking [33]. Moreover, a previous study reported that exposure to cigarette smoke may increase antigen susceptibility and reduce the threshold for antigen sensitization [34]. Cigarette smoke extract (CSE) administration during the sensitization can promote mouse allergic sensitization and the development of an asthma phenotype, and IL-23 may be play an important role in this process [35]. In addition, nicotine can provoke a normal Th2 response, which is more likely to lead to the development and aggravation of asthma [36]. Most importantly, cigarette smoking and asthma interact to trigger certain adverse effects on clinical, therapeutic and prognostic outcomes [37]. Quitting smoking can improve lung function and symptoms, but the low rates of smoking cessation emphasize the need for improved strategies for managing these patients.

In addition, the prevalence of asthma significantly differs according to different regions in mainland China. The prevalence of asthma in East China, North China, Northeast China, South Central China, and Southwest China was 1.87%, 2.68%, 1.57%, 3.16%, 1.87% and 2.50%, respectively. The prevalence of asthma in North and Northwest China was 1.52–1.71 times higher than that in East China, which may be caused by environmental differences. Artemisia pollen is one of the most common inhalant allergens in China [38], especially in the north [39]. Studies have found that Artemisia pollen can trigger autumnal asthma in individuals in northern China [40]. In addition, the level of ambient air pollution during the central heating period in northern China may be related to the high prevalence of asthma [41]. Therefore, the disease burden modification of Artemisia pollen and heating should be taken into consideration ﻿when planning intervention measures to reduce the risk of asthma in northern China.

Another notable risk factor was middle-school education and below, which was significantly correlated with the risk of asthma in accordance with previous studies [7, 15]. With lower education attainment, the prevalence of asthma increased. Compared with those who graduated from high school and above, the risk of asthma was 1.88 times higher for those who graduated from middle school, 2.48 times higher for those who graduated from elementary school, 2.53 times higher for those who were literate and 2.78 times higher for those who were illiterate. In fact, there were higher rates of smoking and obesity, reduced consumption of fruits and vegetables, and higher consumption of saturated fats in those with low education than in those with high education [42, 43]. Thus, people with lower education were more likely to exhibit poor health behaviours (e.g., high BMI) and be exposed to pollution (e.g., cigarette smoke), thus increasing their risk for asthma [44].

Conflicting results have been reported regarding the association of sex with asthma prevalence [7, 45]. A survey from three cross-sectional samples in Sweden showed that the increase in asthma was most pronounced among women from 1996 to 2016 [45]. However, the CHP study showed that the asthma prevalence did not differ between males and females, consistent with our study. The reason may be the increased prevalence of smoking in males in China. We also observed that living in rural areas did not provide protection from asthma, in accordance with other studies [46, 47]. In fact, the difference in asthma prevalence caused by living in urban and rural areas was still connected with the environment. There was sufficient evidence to suggest that air pollutants, such as ozone and particulate matter, trigger exacerbations of asthma, decrease lung function and increase rates of asthma [48]. The result may be masked by the stronger effect of smoking [7] or indicate that a rapid decrease in air pollution is already occurring in China.

The univariate results in this survey demonstrated that the prevalence of asthma was higher in individuals who used unclean cooking fuels than in those who used clean cooking fuels. However, multivariate regression analysis suggested that cooking fule type was not truly relevant to asthma, consistent with the CHP study [6]. Actually, this analysis also observed that the asthma risk associated with biomass use might be masked by the stronger effect of smoking [7], which needs further research for confirmation.

This study has the following advantages. First, the CHARLS is a nationally representative survey targeting populations aged 45 and older from 450 villages or communities in 150 counties or districts in 28 provinces of mainland China, which enabled us to estimate the prevalence of asthma in this age group. Second, this study adopted multistage cluster sampling in six areas of mainland China (East China, North China, Northeast China, Northwest China, South Central China and Southwest China), which avoided selection bias. Third, strict quality control ensured high quality and the reliability of the findings. This study also has several limitations. First, we did not include individuals younger than 45 years. Thus, we cannot provide information on those aged < 45 years old. On the other hand, asthma was identified by self-reported physician diagnoses rather than spirometry measurements, but the study results still reflected the prevalence of asthma in mainland China.

## Conclusions

Asthma is prevalent but underappreciated among middle-aged and elderly people in China. The risk factors for asthma include the race, age, education level, smoking status and residency in North or Northwest China. These results can help to formulate correct prevention and treatment measures for asthma patients.

## Data Availability

The datasets used and/or analysed during the current study are available from the corresponding author on reasonable request.

## Abbreviations

CHARLS: China Health and Retirement Longitudinal Study
WHS: World Health Survey
GINA: Global Initiative for Asthma
GBD: Global Burden of Disease
CARE: China Asthma and Risk factors Epidemiologic
CHP: China Pulmonary Health
CDC: China Center for Disease Prevention and Control
NHIS-NSC: National Health Insurance Service-National Sample Cohort
